# Aerosol transmission of infectious diseases in poultry: microbiology, pathogenesis, and control measures

**DOI:** 10.3389/fmicb.2026.1886873

**Published:** 2026-07-21

**Authors:** Suresh Burlakoti, Anh Dang Trieu Do, Adnan Alrubaye

**Affiliations:** 1 Center for Excellence for Poultry Science, University of Arkansas, Fayetteville, AR, United States; 2 Cell and Molecular Biology Program, University of Arkansas, Fayetteville, AR, United States

**Keywords:** aerosol transmission, control measures, infectious disease, microbiology, pathogenesis, poultry

## Abstract

The expansion of intensive poultry production improves productivity but increases the risk of disease transmission. In poultry houses, infectious disease transmission occurs through direct and indirect routes, including surface contamination and vector-borne transmission. Typically, disease control in poultry mostly focuses on direct contact and management-related practices in poultry houses, but there is growing evidence highlighting the significance of the aerosol transmission route – an indirect route of pathogen spread that can impair production performance in poultry. High stocking density in poultry houses, inadequate ventilation, and poor biosecurity measures increase the risk of aerosolized transmission of bacterial, viral, and fungal pathogens. The efficiency of this route depends on particle size, humidity, temperature, and ventilation, which influence pathogen survival and exposure, and is further compounded by environmental stressors and the site of action within the animal’s respiratory system. Given that major pathogens, such as avian influenza virus, infectious bronchitis virus, avian orthoavulavirus 1, and *Aspergillus* spp., are usually transmitted via the aerosol route, it is essential for poultry farmers to adopt a range of mitigation strategies to achieve effective disease prevention and improve production output. As such, this review highlights the role of aerosol transmission in poultry health and management, focusing on microbiological aspects, pathogenesis, overall impact, and mitigation strategies. Also, this review aims to integrate current knowledge on aerosol characteristics with transmission dynamics and to provide insight into future research opportunities.

## 1 Introduction

In recent decades, the poultry industry has expanded and shifted toward intensive production systems to meet growing consumer demand driven by global population growth and economic development. In the United States, broiler production increased by 17.3% from 2015 to 2024 and is projected to continue on this trajectory in the near future (USDA, 2025), highlighting the growing intensity of the poultry industry nationwide. This ongoing advancement in production systems has led to large flock sizes, rapid flock turnover, and intensive housing and feeding systems (Chivarov et al., 2023; Kistanova et al., 2024). Unfortunately, while production efficiency is improved significantly in such a system, the animals also experience increased exposure to infectious diseases (Gržinić et al., 2023). The rapid expansion of intensive, large-scale poultry farming has necessitated high stocking densities, yet the housing environment is often poorly ventilated, creating favorable conditions for infectious diseases to proliferate. Respiratory pathogens spread rapidly in intensive production systems, and particulate matter (PM) is continuously generated by biological and environmental sources, facilitating the transmission of infectious diseases (Wang et al., 2023; Guo et al., 2022). High-density production poultry houses further increase this risk of pathogen transmission by recirculating air within the flock in a confined environment (Yang et al., 2025).

Current poultry disease control strategies often highlight direct contact transmission, fomite contamination, and water, feed, or litter-borne exposure pathways for infectious bacterial, viral, and fungal pathogens (Kovács et al., 2025; Cambra-López et al., 2010). However, much evidence indicates that airborne contamination plays a vital role in the epidemiology of several viral pathogens, such as infectious bronchitis virus (IBV), avian influenza virus (AIV), avian orthoavulavirus 1 (AOAV-1, previously known as Newcastle disease virus; NDV), Marek’s disease virus (MDV), and other respiratory pathogens (Ssematimba et al., 2012; Jonges et al., 2015; Zhao et al., 2014). Studies suggest several pieces of evidence for the airborne transmission of respiratory infection. A study by Jegede et al. (2019) shows that aerosol transmission of birds with low pathogenic AIV produced higher viral titers, in addition to tracheal and lung cytokine responses, than intranasal or oral exposure, indicating that aerosol is an efficient route for pathogenic infection. Furthermore, AOAV-1 has been detected in aerosols generated by infected birds under experimental conditions (Li et al., 2009). Transmission through the aerosol route is also not limited to viral pathogens. It has been documented that aerosol exposure of avian pathogenic *Escherichia coli* induces respiratory infection and lesions, implicating the role of this transmission route in colibacillosis (Paudel et al., 2021; Kromann et al., 2021; Nguyen et al., 2022). Likewise, other respiratory pathogens – such as *Mycoplasma* spp., *Aspergillus* spp., *Pasturella* spp., and *Avibacterium* spp. – have also been reported to spread through aerosol exposure in poultry houses (Blackall and Soriano-Vargas, 2013; Woo and Kim, 2006; Arné et al., 2011; Landman et al., 2004).

Several pathogens are transmitted via the aerosol route, and their effects on the respiratory system are further influenced by management factors in poultry houses. Collectively, aerosol transmission consists of a broad host-pathogen-environment interplay that involves not only the movement and characteristics of pathogens, but is also shaped by environmental conditions such as temperature, humidity, airflow, and ventilation conditions, which influence the infectivity, pathogen survival, and host susceptibility (Ganapathy et al., 2005; Li et al., 2025; Si et al., 2013). The avian respiratory system is efficient in gas exchange with its rigid lungs, air sacs, and continuous unidirectional airflow, which allows inhaled particles to penetrate deeply and deposit in respiratory tissues (Brown et al., 1997; Fedde, 1980; Maina, 2015). At the same time, dust particles, endotoxin and ammonia within the poultry housing environment can worsen respiratory integrity, change the lung microbiota, and alter inflammatory responses, thereby exacerbating infections from deposited pathogenic particles (Wang et al., 2023; Meijerink et al., 2021; Samy and Naguib, 2018; Zhou et al., 2023). In addition, impaired respiratory function may increase disease susceptibility, resulting in worsened disease state from subsequent coinfections (Samy and Naguib, 2018; Weerts et al., 2021). Together, these findings show that aerosol-related disease risk is determined by both the pathogen and its interactions with environmental stressors and host defenses.

Despite clear evidence in current literature, the impact of aerosol transmission on poultry health requires further studies to address practical on-farm implications. Studies covering this subject tend to focus on respiratory health, pathogen detection, air quality, or mitigation strategies as separate components rather than a holistic, integrated approach to disease transmission. As a result, the available information is diverse, yet scattered across various research areas and disciplines – such as avian pathology, microbiology, engineering, and environmental sciences – making it difficult to gain a clear understanding of aerosol transmission in poultry systems. Therefore, a clear synthesis of aerosol generation, pathogenesis, host exposure, environmental persistence, and control measures is needed in a single framework. This review thus emphasizes current studies on aerosol transmission of infectious diseases in poultry and their implications for bird health and management. More specifically, it focuses on aerosol generation in poultry environments; major pathogens via the aerosol route; pathogenesis and host response; the roles of ventilation and environmental conditions in influencing pathogen survival; and various monitoring, mitigation, and biosecurity strategies for aerosol transmission.

## 2 Aerosol transmission

### 2.1 Concept and definition

**Figure 1:**
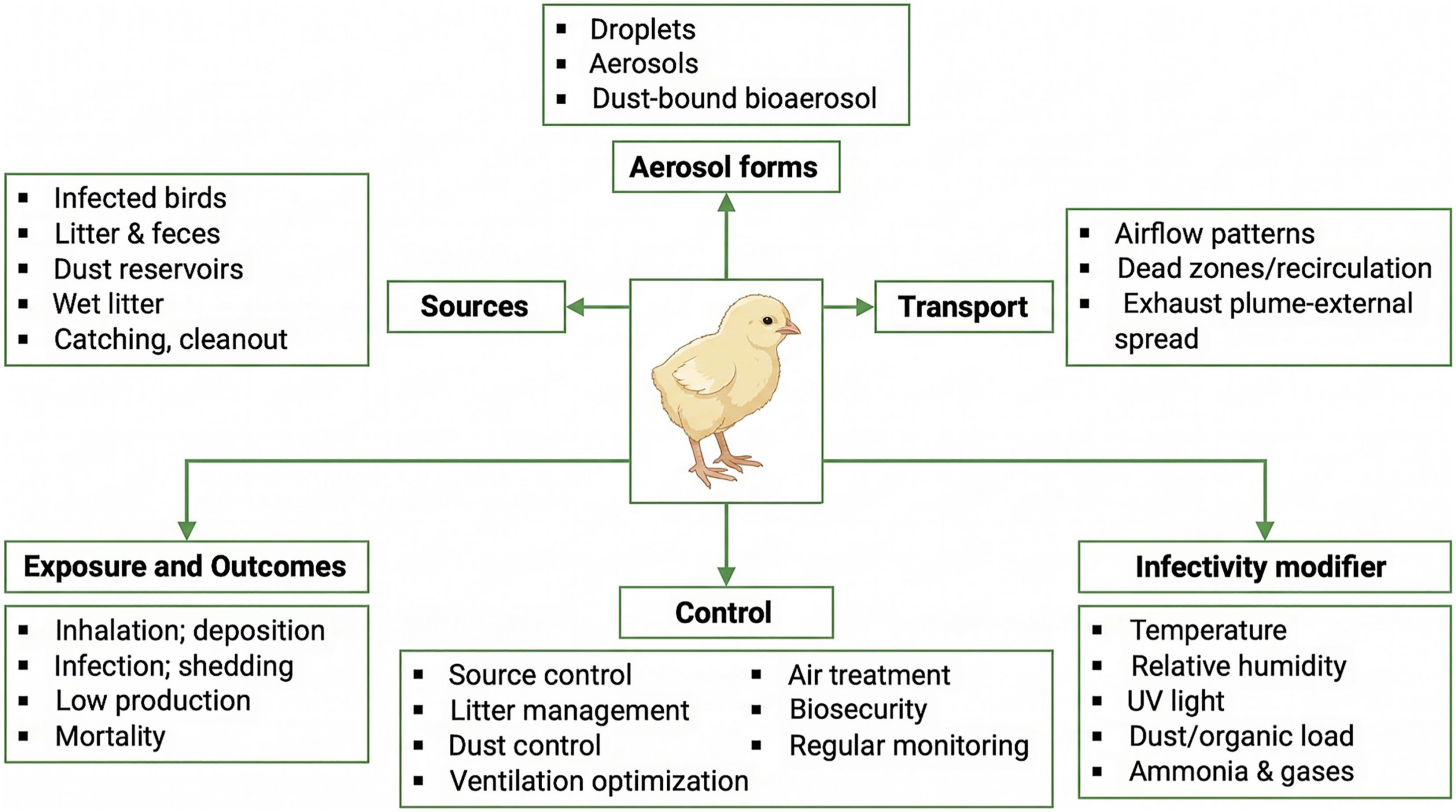
Aerosol transmission pathway summary in poultry houses. This illustration summarizes the sources of aerosols, aerosol forms, aerosol transport, modifiers that reduce or enhance pathogens’ infectivity during transmission, exposure to birds, and disease outcomes. Furthermore, it highlights the overall control strategies for mitigating aerosol transmission on poultry farms.

Aerosol transmission refers to the spread of pathogens via airborne particles inhaled and deposited in the respiratory tracts of birds. It is an important route for the spread of infectious diseases in poultry production, including respiratory and certain enteric pathogens. These pathogens consist of both infectious particles derived from birds and a complex mixture of dust, organic matter, endotoxins, microorganisms, and gases adsorbed onto particulate matter, which can remain suspended in the air and spread within and beyond the housing environment (Wang et al., 2023; Just et al., 2009). In general, pathogenic aerosol transmission depends on particle size and environmental factors such as temperature, humidity, airflow, and ventilation (Pereira et al., 2023; Wang et al., 2025). The particle size of pathogens generally ranges from fine particles (<5 μm) that can travel aerobically over longer distances to large droplets (>10 μm) that tend to settle near the source (Chen et al., 2021). Particle size determines the site of deposition in the respiratory tract and the pathogen’s spread in the housing environment. Larger particles generally deposit in the upper respiratory tract, whereas particles below 5 μm are considered more hazardous, with those below 2.5 μm capable of deeper penetration and may remain in the air for longer periods. James et al. (2023) showed that virus particles below 5 μm show high settling velocities with high concentration in short-range transmission, whereas smaller particles below 2.5 μm form persistent aerosol and carry over long distances to initiate new outbreaks. A summary of aerosol transmission is presented in Figure 1.

### 2.2 Generation and microbiology of aerosol transmission

In poultry housing, aerosols are continuously generated by bird activity, litter, and routine management. Bird respiration and vocalization facilitate the release of fine droplets and respiratory particles, which are then attached to manure dust, feathers, and small feed particles, leading to an airborne load. Litter contributes as the most important source of dust in the aerosolized material, containing biologically active organic compounds from birds’ skin, feathers, and feces, as well as from microorganisms such as viruses, bacteria, fungi, and endotoxin (Cambra-López et al., 2010; Just et al., 2009). In commercial poultry houses, litter is often reused across multiple flocks to reduce production costs, thereby increasing the accumulation of potentially pathogenic materials and the risk of transmission between flocks, particularly when litter windrowing is inadequate or improperly performed (Tabler and Wells, 2017; Hawkins et al., 2022). The levels of environmental contaminants in the dust are also greatly influenced by the associated housing system. Traditional floor housing commonly uses litter materials such as rice hulls or wood shavings, which accumulate feces, feathers, debris, feed residues, and microorganisms that are then recirculated in the air with bird movement. Studies have shown that total dust exposure is higher in floor housing than cage housing systems (Just et al., 2009; Wathes et al., 1997; Kirychuk et al., 2006), with dust concentration at four to five times higher in the former (Ellen et al., 2000). Accumulation of dust in farms also depends on the ventilation conditions. Poor ventilation can lead to high relative humidity, which may reduce dust aerosolization, indicating dust levels are influenced by humidity within the housing environment (Górny et al., 2023).

Dander from chick down and feathers, which naturally increase as birds age with frequency of activities, along with litter-driven organic material such as feces, are also major contributors to aerosol burden (Pereira et al., 2023) as are feed particles from handling, bird consumption, and spillages. Fecal matter can also make up an important source of aerosol microorganisms, with several bacterial species (such as *Salmonella* spp.*, Escherichia coli*) and viruses (such as AIV, IBV) harbored in the feces and attached to fine dust, which eventually becomes part of the burden load (Zhao et al., 2014). This mixture of feed, feather, and fecal particles forms a biologically complex aerosol that carries multiple pathogens, allergens, and inflammatory components (Yang et al., 2025; Zhao et al., 2014). Overall, aerosol generation is associated with a combination of material source, bird activities, housing system, manure and litter management, and the environment created by ventilation and space in the poultry house.

## 3 Pathogenesis and host response to aerosol exposure

**Figure 2:**
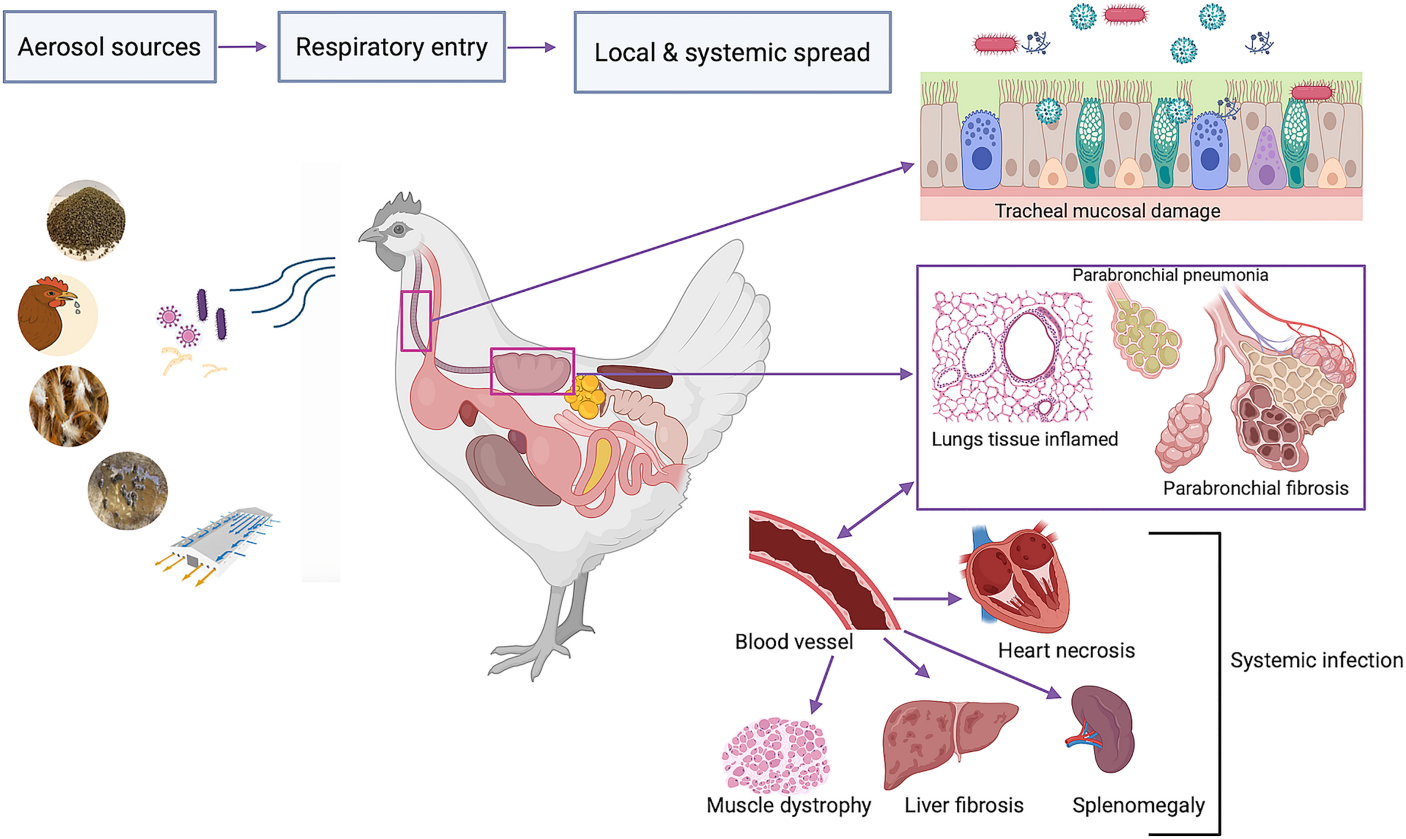
Pathogenesis of aerosol from respiratory entry to systemic dissemination. This figure shows the overall pathogenesis of aerosol-transmitted pathogens, from initial entry into the respiratory tract, local infection in the trachea, infection in the lungs and air sacs, and circulation to all organs of the birds via the blood vessels, causing systemic infection. Created with BioRender.com.

The pathogenesis and host response to aerosolized pathogens are influenced by pathogen transmission to the respiratory tract, interactions with environmental stressors such as dust and ammonia, and effects on the immune system, all of which may impact aerosol-mediated infectious disease (Lou et al., 2022). This results in localized infection of the respiratory tract and lungs, which can then disseminate through the bloodstream and become a systemic infection in the birds, as visualized in Figure 2. This process can be influenced by multiple factors, including pathogen characteristics (type, variants, and species), host status, and the surrounding environment (Isham et al., 2023). Wang et al. (2023) discussed the overall pathogenesis of PM into 3 steps: (1) inhalation of the pathogen irritates the respiratory tract; (2) compromises immune functions; and (3) causes the respiratory infection. This study also highlighted that PM induces respiratory disease via toxin-associated mechanisms such as ammonia ingestion, lung flora dysbiosis, oxidative stress, and metabolic disorder. As interactions involving aerosol transmission and its associated factors (host, environment) are complex and multifaceted, it is essential to investigate each in detail to better understand the underlying mechanisms, on which efficient control measures can be developed.

### 3.1 Respiratory deposition and initial infection

The poultry respiratory system is a structurally complex and functionally efficient gas exchanger with the lung-air sac system: the lung serves as the gas-exchanging part, and the air sac serves as the ventilator (Brown et al., 1997; Fedde, 1980; Maina, 2015). Unidirectional airflow between the bronchi improves the gaseous exchange but is more susceptible to infection (Ali, 2020). The defense mechanisms in the respiratory tract are distinct: some inhaled material is removed in the nasal passages, trachea, and larger bronchi by mucus and cilia, while the remaining particles penetrate the parabronchi and air sacs (Mensah and Brain, 1982). Deposition of inhaled particles largely depends on the particle size, as those smaller than aerodynamic diameter 10 μm commonly deposit in the upper respiratory tract, and those smaller than 2.5 μm in the lower respiratory tract (Wang et al., 2023). Similarly, virus-laden particles smaller than 5 μm deposit in lung tissue where virus replication occurs, then enter the bloodstream and spread to other organs (Tellier, 2006). The study of PM indicates that aerosol exposure is a concern for both the pathogen and its effects, such as inflammation, oxidative stress, and impaired mucosal and immune responses. Wang et al. (2023) pointed out the evidence related to PM in poultry house with oxidative stress, lung flora dysbiosis, and decreased the antioxidant activity in the lung. Zhou et al. (2023) found that PM exposures increase the pulmonary microbiota related to respiratory tract disease and cause chronic inflammation. Another study by Zhou et al. (2024) elaborated that PM2.5 (fine particulate matter with an aerodynamic diameter ≤ 2.5 μm) changes the gut microbiota toward dysbiosis with concurrent development of lung inflammation injury, highlighting the impact on the broilers gut-lung axis. In a similar fashion, Liu et al. (2024) demonstrated that gut microbiota could be affected by PM2.5, inducing lung inflammation in broilers by antigen-presenting cells to activate T- cell differentiation.

In birds, an experimental aerosol model of avian pathogenic *E. coli* (APEC) showed aerogenous infection in adult broiler breeders, with minimal lesions in the trachea and rapid infection in the lungs and air sacs (Kromann et al., 2021). The lesions discovered in this study – including parabronchial impaction and air sac lesions such as bronchitis, pneumonia and airsacculitis – may suggest a weaker immune defense mechanism in the air sacs than in the upper respiratory tract. In APEC studies investigating severity of infection from different exposure routes at equivalent doses, aerosol exposure also causes more severe colibacillosis lesions than intranasal or intratracheal infection (Paudel et al., 2021).

### 3.2 Innate and adaptive immune response

The immune system of birds is complex, involving multiple organs and systems that work together in a coordinated manner. The innate and adaptive immune responses play crucial roles in aerosol infection by influencing pathogen recognition, host-pathogen interactions, and disease outcomes. However, the strength and type of immune response can vary among individual birds and are strongly influenced by age. In young birds, immunity relies on maternal antibodies and the innate immune system, whereas older birds rely more on adaptive immune responses mediated by T and B cells (Meijerink et al., 2021). Major organs of the lymphatic system include the bursa of Fabricius and the thymus – respectively responsible for the development and maturation of B and T lymphocytes – while peripheral immune organs include the harderian gland (HG), paranasal gland (PG), conjunctiva-associated lymphoid tissue (CALT), bronchus-associated lymphoid tissue (BALT), spleen, and caecum tonsil (Rumińska et al., 2008). These organs are directly or indirectly involved in the immune response of the respiratory mucosal system. Upon pathogen inhalation, the lining epithelial cells serve as the first line of defense, with HG, PG, and CALT providing local immune responses within the upper respiratory tract (Phalipon et al., 2002; Smiałek et al., 2011). This defense involves the mechanical removal of the pathogen by epithelial movement and activation of B and T lymphocytes and production of immunoglobulins, which are transported to the nasal cavity and coat the proximal upper airway mucosa (Manz et al., 2005). At the mucosal surface, these immunoglobulins can bind and neutralize inhaled pathogens, and limit their attachment to epithelial cells, which facilitates their clearance from the respiratory tract. Secretory immunoglobulin A (IgA) is particularly important in this mucosal barrier because it helps protect against respiratory infections by providing local immune protection and may enhance immune response following mucosal vaccination.

The route of exposure and vaccination both strongly influence disease pathogenesis and host immune response. Birds inoculated with aerosolized LPAI (low pathogenic avian influenza) had significantly higher virus titers in the trachea and lungs, with higher mortality than those inoculated intranasally or orally (Jegede et al., 2019; Scott et al., 2018). These studies show that aerosol infection with a viral pathogen can induce dysregulated immune responses, producing high levels of cytokines in the lungs, leading to inflammation, tissue damage, impaired lung functions, which manifests in symptoms such as dyspnea, respiratory rales, and respiratory distress, and impacting production performance. However, several studies have shown that administration of anti- AOAV-1 vaccine via intraocular or intranasal routes increases production of antigen-specific IgA (Ganapathy et al., 2005; Russell and Ezeifeka, 1995). Similarly, Bagheri et al. (2023) showed that aerosol vaccination of irradiated APEC induced local immunity, with vaccinated birds showing milder lesions, lower bacterial burden, and a distinct T-cell response. The author further reported that enhancement of adaptive immunity is due to the induction of pathogen-specific IFN-*γ*^+^ CD8α^+^ and IFN-γ^+^ TCR-γδ^+^ T cells in the lungs’ mononuclear cells. These findings indicate that aerosol immunization enhances the cell-mediated immune response essential to the protection of respiratory tissue against the inhaled pathogen.

### 3.3 Interaction with stressor

Generally, infection severity – including those from pathogenic aerosol exposure – can become exacerbated with additional environmental and management stressors on the animal. The most common stressors in poultry production include ammonia, dust, and heat, which can elevate corticosterone levels, weaken the respiratory barrier, increase inflammation, alter the lung microbiome, and impair the defense mechanisms against pathogens (Wang et al., 2022; Dominguez et al., 2023; Chen et al., 2021; Li et al., 2020; Liu et al., 2020; Shini and Kaiser, 2009).

Air quality modulates the immune system and affects disease susceptibility. As an example, ammonia exposure in combination with an aerosolized pathogen can cause more severe damage to the respiratory mucosa. Exposure to ammonia may increase Proteobacteria levels within the lung microflora due to the fluctuation in inflammatory cytokines (IL-1β) and neurotransmitters (5-HT) (Wang et al., 2022), creating more favorable niches for inhaled aerosolized pathogens. These niches allow these pathogens – which have already disrupted the ciliary epithelium of the trachea from the primary infection (Hoerr, 2021) – to persist longer, leading to subsequent infection of the lung and air sacs, and compromising the immune function. Compounding with ammonia exposure, studies have shown that heat stress impairs lung functions by damaging the pulmonary blood-air barrier, increasing lung permeability, and altering the inflammatory response, which in turn leads to reduced immune functions and defense against the inhaled pathogen (Brugaletta et al., 2022). With improper house management, excessive ammonia levels at elevated temperatures in the farm can alter physiological processes in the birds, further suppressing immune functions and making birds more prone to aerosol infections. Therefore, good housing management practices are necessary to reduce the severity of pathogen infection and mitigate the risk of serious, yet preventable, health impacts.

As a familiar concept in pathology across many host species, co-infections also constitute another factor that worsens the disease state in aerosol infections. Hassan et al. (2017) reported that co-infection with IBV and AIV-H9N2 induced severe clinical signs and symptoms with high mortality and, in experimentally infected birds, increased the shedding of AIV-H9N2 in IBV co-infection. Kong et al. (2021) findings showed similar results as IBV and AIV-H9N2 co-infection exacerbate respiratory disease severity with high morbidity and mortality when IBV infection precedes AIV-H9N2 exposure. More recently, Huang et al. (2024) demonstrated that AIV-H9N2 and IBV co-infection increased mortality, showed clinical lesions, decreased growth performance, and enhanced H9N2 replication, which may be mediated through dysregulated immune and metabolic pathways. Furthermore, studies have shown that after respiratory infection of IBV and AOAV-1, more severe tracheitis and airsacculitis were observed than with infection of APEC alone (Weerts et al., 2021; Kathayat et al., 2021). This indicates that prior damage to mucosal defenses and suppressed immune function facilitate the proliferation of secondary bacterial infections.

In most cases, proper vaccination suppresses infectious disease and boosts the immune system against infections; the opposite can occur with heightened vaccination stress to intensify the pathogenesis of aerosol pathogens, depending on the bird’s condition and other environmental factors. Improper vaccination under respiratory stressors, including ammonia and dust, poor ventilation, and concurrent infection, intensifies aerosol-transmitted pathogen infection by enhancing physiological stress and reducing mucosal immune defenses. A study on Newcastle disease vaccination showed that the magnitude of stress and acute-phase protein responses after vaccination varies by vaccine type and regimen, indicating that vaccination can induce short-term physiological stress while simultaneously activating immune responses (Janmohammadi et al., 2020). Nielsen et al. (2023) reported that HPAI (highly pathogenic avian influenza) vaccination can reduce pathogen transmission, but its efficacy varies depending on the vaccine strain and its match to the circulating strain. In contrast, a study by Song et al. (2020) found that a high frequency of vaccination for AOAV-1 induces immune stress. Paradoxically, Li et al. (2020) suggested that vaccination stress can upregulate stress hormones and – ironically – suppress immune functions, compromising the host against pathogens. These examples highlight the importance of robust, effective, and high-compliance vaccination regimens for optimal protection against aerosolized pathogens and diseases, without risking undue adverse effects from overreliance on vaccines, thereby improving overall flock health and productivity.

## 4 Major aerosol transmitted pathogen in poultry production

Several major bacterial, viral, and fungal pathogens are associated with the aerosol transmission route in poultry production, causing significant economic losses annually to the poultry industry. Table 1 summarizes the overall pathogen transmission via the aerosol route.

**Table 1:** Aerosol-associated major pathogens in poultry.

Pathogen	Pathogen type	Disease	Sources	Evidence for aerosol	Outcomes	References
Infectious bronchitis virus	Virus	Infectious bronchitis	Respiratory droplets; aerosol	Rapid within flock spread via aerosol; also, via indirect routes	Respiratory signs and symptoms; performance losses, reproductive lesions	Cavanagh (2007); Falchieri et al. (2024)
Avian orthoavulavirus 1	Virus	Newcastle disease	Respiratory aerosol	Infection via aerosol-exposed birds	Respiratory or systemic infection; mortality varies by strain	Li et al. (2009); Hugh-Jones et al. (1973); Hostyn et al. (2025); Sinha et al. (1957); Schiappacasse et al. (2020)
Avian influenza virus	Virus	Avian influenza	Dust; droplets; aerosol	Detection in air/dust at and beyond farms	Respiratory/systemic signs; high morbidity and mortality, major economic loss	Jonges et al. (2015); Filaire et al. (2022); Mahmoud et al. (2024); Scoizec et al. (2018)
Marek’s disease virus	Virus	Marek’s disease	Feather dander/dust	Infection via inhalation of infectious dander	Tumors, immune suppression, production impacts	Atkins et al. (2011); Mulyati et al. (2025)
*Mycoplasma* spp.	Bacteria	Mycoplasmosis	Aerosol transmission	Infection via generated aerosol	Respiratory distress and infections; synovitis	Landman et al. (2004); Marouf et al. (2022); Mugunthan et al. (2023)
*Avibacterium paragllinarum*	Bacteria	Infectious coryza	Short range droplets	Spread via droplet and contact	Respiratory; oculo-nasal infection, drop in egg production	Blackall and Soriano-Vargas (2013); Beach and Schalm (1936); El-Gazzar et al. (2025)
*Escherichia coli*	Bacteria	Colibacillosis	Dust; droplet; aerosol	Spread via dust, manure, and water	Respiratory distress, pneumonia	Kromann et al. (2021); Nguyen et al. (2022); Bagheri et al. (2023); Khairullah et al. (2024)
*Pasturella multocida*	Bacteria	Fowl cholera	Respiratory droplet; aerosol	Spread via respiratory secretion	Respiratory infection	Woo and Kim (2006); Simensen and Olson (1980)
*Aspergillus* spp.	Fungus	Aspergillosis	Aerosolized spores form litter/feed	Inhalation of spores; affected by dust and ventilation condition	Air sacculitis, pneumonia	Arné et al. (2011); Munir et al. (2017); Alhassani et al. (2025)

### 4.1 Bacterial pathogens

As previously mentioned, bacterial pathogens are transmitted via aerosols in poultry production systems, with dust, particulate matter, and droplets serving as carriers. Poultry houses provide favorable conditions for bacterial aerosols due to high stocking density, litter-driven dust, excessive bird movement, and mechanically enclosed spaces, thereby enhancing the generation and circulation of pathogens. Aerosolized bacterial transport is influenced by particle size, as particles remain suspended and travel to different regions of the respiratory tract. Li et al. (2021) reported that particulate matter (PM) ranging from 2.1 to 4.7 μm can facilitate the transport of bacterial aerosols that cause oxidative stress responses and impact respiratory immune functions. Furthermore, the composition of bacterial aerosol is shaped by production systems, bird age, ventilation, and climate conditions. Evidence shows that bacterial aerosol burden changes over production cycles, with Wang et al. (2025) reporting that airborne bacterial aerosols in winter broiler houses increased alongside birds’ age, with bacterial concentration increasing from 1.1 × 10^3^ to 1.6 × 10^4^ CFU/m^3^ between 7, 21, and 35 days of age, while particle size reduced from ≥4.7 μm at day 7 to <4.7 μm at later stages. Bacterial diversity and richness also increased with age, with predominant genera including *Stenotrophomonas*, *Lactobacillus*, and *Ruminococcus* (Wang et al., 2025). Similarly, Asnayanti et al. (2024) evaluated an aerosol transmission-based bacterial chondronecrosis with osteomyelitis (BCO) induction model in broilers. In this model, seeder birds reared on wire flooring (Wideman et al., 2012) served as a source of etiological agents for birds reared on litter flooring within the same housing environment. In addition to the clinical lameness observed in the pure wire-flooring induction model, the disease was also successfully transmitted from seeder birds to littermates in the aerosol transmission model, highlighting the role of ventilation-driven aerosol transport of bacterial pathogens within the same environment, similar to horizontal transmission commonly experienced in commercial poultry production (Kovács et al., 2025). Together, these findings indicate that the bacterial composition of aerosols can be influenced by surrounding environmental conditions, and that infection resulting from exposure to pathogenic aerosols can indeed reproduce similar disease conditions in infected individuals. Such insights are useful to understand disease pathogenesis and the development of preventive approaches against bacterial pathogens in the production environment.

Some of the most prevalent aerosol-transmitted bacterial pathogens include *Mycoplasma* spp.*, Avibacterium paragallinarum*, *Escherichia coli*, and *Pasteurella multocida*. Mycoplasmosis is caused by the bacterial pathogen *Mycoplasma* spp., which is among the most important aerosol-spread bacterial respiratory pathogens. *Mycoplasma gallisepticum* infection causes chronic respiratory disease and infectious sinusitis, resulting in pathological changes such as conjunctivitis, sinusitis, tracheitis, and airsacculitis and significant economic losses from reduced weight gain, decreased egg production, and poor feed efficiency (Marouf et al., 2022; Mohammed et al., 1987; Mugunthan et al., 2023). On the other hand, *Mycoplasma synoviae* causes infectious synovitis, lameness, comb atrophy, eggshell apex abnormalities, and mild upper respiratory infection (Catania et al., 2016; Kleven, 2008; Landman and Feberwee, 2012). The severity of aerosol-transmitted mycoplasmosis is also highly dependent on the respective associated species. A study by Landman et al. (2004) found that *M. synoviae* aerosolized under experimental conditions; however, its viability was lower compared to *M. gallisepticum* under the same conditions. This suggests that airborne transmission of *M. synoviae* – while possible – may bear lower persistence, thus limiting its survivability, potentially reducing infectivity.

Infectious coryza, caused by *Avibacterium paragallinarum*, is another aerogenous disease of high interest to the poultry industry. It is primarily associated with short-range droplets and direct contact rather than long-distance airborne transmission. Infectious coryza causes upper respiratory infections in chickens and shows symptoms of ocular-nasal and upper respiratory infections (Blackall and Soriano-Vargas, 2013; Beach and Schalm, 1936). Clinical signs such as edema of the face and wattle also show in infected birds regardless of age (Blackall and Matsumoto, 2003). It affects production by causing high morbidity and mortality (particularly in complicated and coinfection cases), including arthritis and septicemia; reduced feed and water intake; poor growth performance in broilers; and egg production decline in layers, resulting in significant economic impacts on poultry production (Han et al., 2016; Blackall, 1999; El-Gazzar et al., 2025).

*Escherichia coli* is also an important bacterial pathogen, with aerosol transmission considered a highly efficient route in the pathogenesis of colibacillosis. Common clinical signs and symptoms include localized respiratory infection to systemic infection, with additional complications such as acute septicemia, peritonitis, pericarditis, perihepatitis, and airsacculitis (Lutful Kabir, 2010; Khairullah et al., 2024). This disease causes severe economic losses because of the decrease in egg production in layers and overall production performances in broilers with high mortality (Joseph et al., 2023). Kromann et al. (2021) developed the aerosol infection model of colibacillosis in broiler breeder using *E. coli* and found typical clinical signs and lesions in multiple organs, both histologically and grossly. This study highlights that airborne exposure can reproduce key pathological features of colibacillosis and provides valuable tools for studying disease mechanisms to develop prophylactic strategies.

Finally, *Pasteurella multocida* is another bacterial pathogen relevant to aerosol transmission. The causative agent of fowl cholera, *P. multocida* is transmitted by respiratory droplets and aerosols shed from infected birds. Inhalation of the pathogen via aerosol can lead to respiratory tract infection in birds, especially in dense housing systems (Woo and Kim, 2006; Simensen and Olson, 1980). Fowl cholera is associated with acute septicemia and complications such as lameness in addition to swollen wattles and comb, which leads to major economic losses through decreased production, high flock mortality, and treatment costs (Carpenter et al., 1988; Christensen et al., 2008). Current evidence indicates that the occurrence and potential for transmission of bacterial aerosols depend on particle characteristics and pathogen-specific biological factors. Therefore, effective management of the housing environment and knowledge about the pathogenicity is essential to control exposure to bacterial aerosols.

### 4.2 Viral pathogens

Aerosol transmission is an important route of viral pathogen spread in poultry houses, especially in high-density flocks and intensive production systems. In viral diseases, virus-laden particles come from contaminated litter, feathers, skin fragments, respiratory secretions, and fluids released during handling, transport, and slaughter of birds (Filaire et al., 2022; Rimi et al., 2025). The primary pathogens include infectious bronchitis virus (IBV), avian orthoavulavirus 1 (AOAV-1), avian influenza virus (AIV) and Marek’s disease virus (MDV). AIV is a highly contagious viral pathogen of poultry that can be transmitted efficiently via aerosols, causing avian influenza (Si et al., 2013; Samy and Naguib, 2018). It causes severe systemic disease characterized by respiratory distress, pulmonary congestion, edema, hemorrhages, and necrosis in multiple visceral organs, and high morbidity and mortality resulting in huge economic loss in the poultry industry (Pantin-Jackwood and Swayne, 2009; Swayne and Suarez, 2000; Ramos et al., 2017; Mahmoud et al., 2024). AIVs are categorized as low-pathogenic avian influenza virus (LPAIV) or highly pathogenic avian influenza virus (HPAIV) based on the pathogenicity (Lv et al., 2021). Like other bacterial and viral pathogens, the virus is shed in respiratory secretions and feces of infected birds, contaminating litter, dust, and feathers, which subsequently become sources of aerosol transmission via particles and large droplets within the housing environment (de Vos and Elbers, 2024). Several studies have documented dust as a major source of AIV contamination through aerosol transmission. Filaire et al. (2022) studied aerosol transmission of the H5N8 strain of AIV in dust samples from a poultry shed and found that contaminated dust may also contribute to viral dispersal in the early stages of infection, hinting at dust particles as a valuable tool for AIV surveillance. Furthermore, Jonges et al. (2015) detected AIV in airborne particulate matter from infected poultry sheds up to 60 m downwind and indicated that contaminated dust may lead to short-distance dispersal of the pathogen. Likewise, Scoizec et al. (2018) detected AIV RNA in air samples inside poultry shed at distances up to 50–110 m downwind and found a decline in viral concentration with distance, indicating airborne dispersal of the pathogen.

In addition to AIV, the infectious bronchitis virus, the coronavirus of poultry, is highly contagious and rapidly spreads in large flocks, affecting production performance. Its transmission occurs through aerosols and direct contact with contaminated materials, and the pathogen replicates in the respiratory tract and alimentary canal (Cavanagh, 2007; Falchieri et al., 2024). The severity of infection of IBV varies with breeds, which is related to the immune response of the birds (Isham et al., 2023). AOAV-1 is another contagious viral pathogen of poultry and causes Newcastle disease. It is transmitted through respiratory aerosols in both open-air and closed housing systems (Hugh-Jones et al., 1973; Hostyn et al., 2025). Various environmental conditions favor increased aerosol transmission and the severity of AOAV-1 infection (Sinha et al., 1957). Li et al. (2009) studied the experimental isolator model for aerosol transmission of AOAV-1 and found that infected birds released AOAV-1 into the air through aerosols and infection was transmitted only to the shared airflow. Aerosol-exposed birds showed viral shedding and seroconversion, implicating that aerosol transmission of AOAV-1 is possible in a controlled environmental condition. Schiappacasse et al. (2020) investigated inactivation techniques using a laboratory-scale, non-thermal plasma reactor for aerosolized AOAV-1. Results from the study found that the pathogen was inactivated, with higher concentrations at lower relative humidity, suggesting that non-thermal plasma is a promising method for controlling aerosol transmission of AOAV-1.

Similarly, MDV is another contagious airborne pathogen in poultry that causes Marek’s disease. Its transmission may also occur via virus-laden feather dander and contaminated dust from infected birds (Atkins et al., 2011; Mulyati et al., 2025), indicating that feather- and dust-associated particles can serve as important vehicles for MDV aerosol transmission in the poultry house. The viral pathogens IBV, AOAV-1, AIV, and MDV differ in their primary routes of aerosol transmission and environmental persistence. IBV spreads rapidly by aerosol, AOAV-1 recovered from aerosol-generated infected birds, AIV associated with short- and long-range aerosol dispersal, whereas MDV sheds the pathogen in feather follicles and dust (Li et al., 2009; Isham et al., 2023; Scoizec et al., 2018; Atkins et al., 2011). Together, these findings indicate that the nature of pathogen transmission can vary with the specific pathogen source, dispersal route, and environmental persistence, necessitating a comprehensive, critical understanding of aerosol transmission and airborne exposure to various pathogens in intensive production systems for effective disease management.

### 4.3 Fungal pathogens

Fungal disease is a significant cause of economic losses in the poultry industry because of the high mortality and morbidity associated with mycotoxins in feed, which have severe toxic effects to broilers (Asfaw and Dawit, 2017). Mycotoxins suppress the immune system, making birds more susceptible to bacterial and viral infections, thereby affecting health and overall performance (Resanović et al., 2009). The main fungal diseases reported in poultry production include aspergillosis, candidiasis, and fusarium-induced osteochondrosis (Hauck et al., 2020; Sharma et al., 1970; Walser et al., 1982). Aerosolized fungal pathogens can also cause fungal diseases and toxin poisoning. In general, fungal aerosols span a broad particle size range, but some particles occur within the PM2.5 and represent a variety of fungal species (Li and Kuo, 1994). Common fungal pathogens in the poultry industry associated with fungal infections include the genera *Fusarium, Aspergillus, Rhizopus, Penicillium, Mucor,* and *Alternaria* (Krnjaja et al., 2010). The dominant species found in fungal aerosols include *Aspergillus*, *Trichosporon, Candida, Cladosporium* and *Alternaria* (Chen et al., 2021).

In particular, *Aspergillus fumigatus* is a major fungal pathogen of significant interest in the poultry industry, alongside other species such as *A. flavus, A. nidulans*, and *A. terreus*. Inhalation of aerosolized *A. fumigatus* conidia (asexual spores of filamentous fungi) from contaminated feed, litter, or bedding (Arné et al., 2011; Munir et al., 2017) causes them to deposit deep in the respiratory tract, resulting in aspergillosis. In the acute form, it causes high mortality and morbidity in young birds, whereas the chronic form generally affects older birds and impacts production performance (Kunkle et al., 2003). Interestingly, Chen et al. (2021) highlighted that fungal pathogen concentration increases with bird age, alongside the variety and diversity of fungal communities in the aerosolized form. This may be correlated with improper house management as the flock ages, leading to dusty conditions (inadequate ventilation) or moist, moldy litter, creating a favorable environment for the growth of fungal pathogens, thereby increasing their generation and transmission in poultry houses (Arné et al., 2011; Kunkle et al., 2003; Alhassani et al., 2025).

### 4.4 Environmental persistence

Once pathogens are aerosolized, their persistence depends on various environmental factors in the housing environment, such as ventilation, temperature, humidity, airflow, and the nature of the carrier particle. Ventilation types and airflow patterns influence aerosol transmission and generation, with several studies hinting at the possibility may reduce aerosol persistence. Adequate airflow, facilitated by effective ventilation, allows for the rapid expulsion of pathogens and generated aerosols, ultimately reducing the risk of infection in animals (Li et al., 2019). In addition, proper ventilation helps to maintain lower ammonia levels and appropriate temperature ranges, which improves birds’ comfort, litter quality, health, and productivity. Better air velocity lowers ammonia emission and enhances welfare and production by reducing aerosol burden (Gonçalves et al., 2023; West et al., 2024). On the other hand, poor ventilation leads to prolonged persistence of pathogens in the housing environment, resulting in increased pathogen load and subsequent transmission (Winkel et al., 2015). Du et al. (2019) has also discovered that tunnel-ventilated houses may develop high bioaerosol concentrations in localized regions at the rear part of the house, depending on airflow distribution. This dual nature of mechanical ventilation in relation to aerosol transmission, either as a control measure or a dispersal mechanism, is highly dependent on effective management of the housing environment, underscoring the need for sound management approaches and strategies by farmers and producers.

Humidity and temperature also significantly impact particle behavior and microorganism survival in aerosols. Li and Kuo, (1994) noted that fungi can grow and produce spore easily in an environment with high relative humidity (60–90%) and mild temperature (15–37 °C). Data from Xu et al. (2022) concurs with these findings, showing positive correlation between environmental factors – such as temperature and relative humidity – and bacterial population growth. Paralleling these studies, Wu et al. (2017) indicated that humidity also strongly influences microbial aerosol behavior, as it varies by season and time of day, with endotoxin levels significantly correlated with humidity, suggesting that moisture levels may enhance the survival, release, and suspension of biologically active aerosol components.

Carrier materials, such as dust particles, litter fragments, feather pieces, and fecal matter, play important roles in the persistence and transport of microorganisms. Nguyen et al. (2022) found that 98.9% of airborne *E. coli* were associated with dust particles with a diameter greater than 2.1 μm. Similarly, Wang et al. (2023) reported that particulate matter can adsorb and transport microorganisms, ammonia, endotoxins and other pollutants, acting as a biological transport medium. This highlights that the carrier particle may facilitate the spread and deposition of microbes while also protecting attached microbes from environmental stressors during transmission. Overall, the aerosol generation in poultry houses results from multiple interacting processes involving birds, litter, and housing conditions and extending to the environment via the ventilation-driven dispersal.

## 5 Mitigation and control strategy

Prevention and control of infectious diseases related to aerosol generation are major aspects of production systems. The principal mitigation strategy includes identifying aerosol pollutant sources, as well as an in-depth understanding of housing designs, ventilation systems, and other important associated risk factors (Guo et al., 2022; La et al., 2022). Understanding the transmission mechanisms of a pathogen is also a principal factor in minimizing its infectious spread, contributing to its effective prevention and control (Van Seventer and Hochberg, 2016). The most relevant mitigation and control strategies for pathogen transmission via the aerosol route are discussed below and summarized in Table 2.

**Table 2:** Mitigation and control strategies for aerosol transmission pathogens.

Categories	Methods	Main purpose	References
Biosecurity	External, internal and operational biosecurity	Prevent entry and spread of pathogens through management, disinfection, PPE, and movement control	Dewulf and Van Immerseel (2019); Dorea et al. (2010)
Vaccination	Aerosol or routine vaccination	Reduce infection, shedding, and airborne spread of pathogens	Peeters et al. (2014); Bertran et al. (2018)
Ventilation	Optimized airflow and ventilation design	Dilute aerosol load and reduce indoor accumulation of particles	Ritz et al. (2006); Mitchell et al. (2004)
Air inlet protection	Filtration or inlet barriers	Reduce entry of contaminated particles and insects from outside	Elbers et al. (2022)
Dust control	Litter management and reduced disturbance	Lower dust associated microorganisms and aerosol generation	Wang et al. (2023); Atapattu et al. (2017)
Ammonia control	Litter amendment and moisture management	Reduce ammonia stress and improve respiratory environment	Atapattu et al. (2017)
Air treatment	UVB/UVGI and ESCS	Inactivate or remove airborne pathogens and dust	Ritz et al. (2006); Sutton et al. (2013)
Nutritional intervention	Antioxidant and immune modulating diets	Improve resistance against aerosol-induced inflammation and infection	Asnayanti et al. (2024); Perera et al. (2024); Shojadoost et al. (2020); Zhang et al. (2023)

### 5.1 Biosecurity and farm management

Biosecurity is a core component of preventive measures and guidelines designed to reduce the risk of pathogenic infection and spread in farm environments (Huber et al., 2022). Strict and sound implementation of proper biosecurity protocols remains essential for disease prevention in any poultry operation as conventional measures primarily focus on direct contact and indirect transmission via fomites, equipment, personnel, wild birds, and other vectors (Kovács et al., 2025; Huber et al., 2022; Joshi et al., 2025). In high-stocking-density commercial poultry production, the importance of proper biosecurity is emphasized even further due to aerosol-transmitted pathogens, as strict biosecurity limits pathogen transmission, supporting production performance and reducing reliance on pharmacological interventions. The main focus is restricting access to the poultry farm and following strict hygiene protocols – including regular monitoring of flock health, management of waste, and feed storage to reduce risk of contamination and generation of aerosol (Grace et al., 2024; Dewulf and Van Immerseel, 2019). Moreover, farm location and site selection should consider the spacing of other poultry houses and other operations, as well as wind direction, which determines the intensity and direction of aerosol transmission. For aerosol-transmitted pathogens, additional measures should be implemented alongside proper farm location and house design, including dust level control, maintaining litter moisture, optimizing ventilation rate with air filtration, or air treatment, and maintaining stocking density to prevent the risk of aerosolized pathogen transmission of infectious disease.

A well-rounded, three-dimensional biosecurity system should fully consider external, internal, and procedural-operational production aspects that are critical to controlling pathogens both outside and inside the housing environment (Kovács et al., 2025; Sims, 2015; Dorea et al., 2010; FAO, 2011). External biosecurity includes preventing the introduction of pathogens from external sources – such as farm equipment, workers, feed, vehicles, wild animals and airborne particles originating from nearby farms or contaminated environments – to the house and animals. To control this, the use of personal protective equipment (PPE), disinfection of all equipment, and prevention of contamination via feed and water are necessary. In contrast, internal biosecurity measures include cleaning and disinfecting facilities following all-in/all-out flock movements, managing stocking densities, culling infected birds, and managing litter in dry conditions but not excessively dusty, feed and water management with ventilation to prevent contamination of aerosols within the house. Finally, procedural-operational biosecurity includes the personal-measures level for the proper implementation of biosecurity practices, such as hand sanitation, footbaths, use of PPE, movement control, and strict adherence to cleaning and disinfection protocols.

### 5.2 Vaccination

Vaccination is an important control method for many aerosol-associated poultry diseases. Its protective efficacy can depend on the route of vaccine administration. Aerogenous vaccine delivery can protect broilers against pathogen shedding and aerosol dissemination in the respiratory tract by enhancing mucosal and systemic immunity (Peeters et al., 2014). A study of AIV has shown that aerosol delivery of the vaccine reduced infection, which is also considered a cost-effective method for controlling AIV (Cargnin Faccin et al., 2025). Bertran et al. (2018) studied airborne transmission of H5N1 HPAI from infected poultry and found a lack of infectious airborne particles in vaccinated chickens during processing of infected poultry, showing a reduction of airborne virus release and transmission during processing with the use of vaccination.

Mucosal vaccination routes, such as through spraying, intranasal, and ocular, are particularly relevant as the primary sites of pathogen entry. In the case of IBV, Deville et al. (2012) found that mucosal delivery of live vaccine improved protection against the pathogen. Bagheri et al. (2023) found that aerosol vaccination with irradiated APEC induced local immune responses against avian-pathogenic *E. coli*. More recently, Wang et al. (2025) found that spraying and the bivalent AOAV-1-H9N2 AIV vaccine protected against viral shedding and lethal virus challenge, the that the route of administration was noted to be useful for mass application. Similarly, Cargnin Faccin et al. (2025) also demonstrated that bivalent H9N2/H5N2 AIV vaccination provided protection against avian influenza of this strain using sequential aerosol and oral vaccination. However, given the associated stress and potential reduction in broilers’ immunity following frequent vaccination, a robust vaccination schedule is essential for effective control of aerosol-mediated diseases. Additionally, a knowledge gap remains in the current literature on multivalent vaccines targeting multiple pathogens. With the current rise in research interest investigating the applicability of various radiation technologies in vaccine development, such as electron beam (eBeam), there is significant room for future growth in the field, ultimately contributing to highly reproducible, sustainable prevention and control of infectious poultry diseases (Perera et al., 2025; Wijewardana et al., 2023).

### 5.3 Engineering, dust, and aerosol control

As previously discussed, Wang et al. (2023) highlighted that bird age, litter condition, and the housing environment are major drivers of airborne contaminants. Therefore, engineering controls, which refer to physical and mechanical intervention strategies (ventilation, air filtration, and airflow management) without relying on pharmacotherapeutic measures, constitute an important aspect of the control of aerosol transmission.

Litter condition management through moisture monitoring and daily husbandry practices is a practical approach for aerosol control in a poultry house. Widely regarded as an effective tool for poultry farmers, litter amendment is one of the most popular practices for controlling litter ammonia, which acts as an inherent stressor for birds in the generated aerosols. Atapattu et al. (2017) studied the impact of two litter amendments, Mizuho-microbial culture mix, and Rydall OE-enzymatic biocatalyst, on ammonia in a closed broiler housing environment and found the enzymatic biocatalyst was more effective than microbial culture in reducing ammonia levels. In the same vein, several other engineering technologies have been investigated, with promising results in reducing aerosol bacterial loads in poultry housing environments. Ritz et al. (2006) found that the electrostatic space charge system (ESCS) significantly reduced airborne dust and ammonia concentration in the house. In agreement, Mitchell et al. (2004) determined that ESCS was able to reduce airborne dust and gram-negative bacteria, and Richardson et al. (2003) also found that ESCS significantly reduced airborne transmission of *Salmonella*, which is reliable and easy to maintain on the farm. Air filtration is another control method for pathogen prevention, in which a filter is placed at the inlet, through which air passes before reaching the birds. Filtration removes dust, droplets, and bioaerosols through an efficient filtration process. A study found that pathogens could enter poultry houses through air inlets and identified *Campylobacter*-positive particulate matter at these entry points (Elbers et al., 2022). Furthermore, effective use of mechanical ventilation can facilitate rapid expulsion of dust and associated pathogens from the housing environment (Du et al., 2019). Evidently, proper litter and ventilation management can reduce dust-associated microbial and ammonia levels, thereby reducing mucosal irritation and injury in birds, ultimately reducing their susceptibility to pathogenic airborne transmission.

In addition to those discussed, Ultraviolet (UV) radiation is another promising technique for controlling pathogens, albeit with significant safety concerns. The UV spectrum B (UVB) is considered environmentally effective for virucidal radiation (WHO, 2012). While the use of radiation can damage the nucleic acid and effectively inactivate the pathogen, this also exposes workers to radiation danger. For example, with the use of UVB in the case of HPAIV and AOAV-1 (Sutton et al., 2013), shielding from radiation exposure is necessary to prevent health hazards for farm workers. In tandem, ultraviolet germicidal irradiation (UVGI) and mechanical ventilation can be considered complementary approaches, as UVGI inactivates viral pathogens while ventilation reduces aerosol load, maintains humidity, and enhances air quality (Nunayon et al., 2026). Many other emerging technologies such as microwave, ionization, non-thermal plasma, and photocatalyst (Ouyang et al., 2023) are also prospective candidates. These technologies eliminate pathogens and contribute to a cleaner, healthier immediate environment through a variety of mechanisms – including air purification, rapid heating to inactivate pathogens, chemical oxidation, or free electrons and radicals – with a wide range of applications that are still in active development and refinement in preparation for commercialized use. Overall, engineering and environmental controls provide a promising strategy for preventing aerosol transmission in poultry houses, with ample room for growth and warranting further extensive research.

### 5.4 Nutritional intervention

Innovative nutritional intervention strategies for the mitigation and control of host-specific aerosol pathogens have become increasingly relevant in the current literature, with practical implications for real-world production settings. Aerosol exposure is a concern because of the associated pathogen and its effects on overall health. Effective nutritional interventions, such as probiotics, prebiotics, phytogenic compounds, vitamins, and minerals, may support resistance to aerosol-associated infectious diseases (Shehata et al., 2022; Alagawany et al., 2020). These interventions can enhance the immune system during infection, regulate leukocyte function, alter endocrine-mediated responses, and maintain gut microbial balance and intestinal integrity (Klasing, 1998). More importantly, effective nutritional intervention strategies can provide consistent, long-lasting benefits throughout the animal’s productive life – often without incurring extensive additional costs from operational and structural changes that may financially burden farmers and producers.

Several nutritional supplements such as probiotics, vitamins, and trace minerals have shown potential for infectious disease control. Asnayanti et al. (2024) studied the dietary supplementation of 1, 25-dihydroxyvitamin D3, which plays an important role in mineral homeostasis, bone health, and immune systems, against aerosol-induced BCO lameness in broiler chicken and found a promising nutritional intervention for reducing the BCO lameness, with 1.0 μg/kg by 54%, and supplementation during the first 28 days provides substantial protection. Similarly, Perera et al. (2024) found that supplementation of an *Enterococcus*-based probiotic in the broiler diet significantly reduced the BCO lameness by 46% in the same experimental aerosol transmission model. Shojadoost et al. (2019) studied selenium supplementation in diets followed by LPAIV and found significantly lower viral shedding, implicating enhanced antiviral defense and control against viral infections. Furthermore, Shojadoost et al. (2020) found that selenium supplementation enhanced vaccine-induced immunity against AIV. These studies highlight the potential commercial use of supplemental selenium against viral infections. Zhang et al. (2023) found that vitamin A supplementation enhances immune function against IBV infection by inhibiting viral replication, increasing antibody titers, and reducing the inflammatory response. These studies suggest that supportive nutritional intervention is beneficial in mitigating aerosol-borne pathogens. Unfortunately, a significant knowledge gap remains in nutritional studies on pathogenic aerosol transmission in poultry compared with those focusing on production aspects and related topics. Therefore, further research on specific nutrients, including their doses and applications, is warranted to support the development of additional nutritional interventions as mitigation and control strategies for aerosol transmission.

## 6 Discussion

Aerosol transmission is an important route for the spread of infectious diseases caused by a wide range of pathogens. Compared to mammals, avian species possess a unique respiratory system with lungs and air sacs and has a unidirectional air flow, whereas mammals involve the upper and lower respiratory tract driven by tidal ventilation (Hostyn et al., 2025; Wang et al., 2021). This anatomical and physiological difference in birds allows transmission, deposition and persistence of aerosolized pathogens to reach deeper into the respiratory tract, including parabronchi and air sacs, compared with mammals. Despite being a significant and relevant route for infectious disease outbreaks in poultry production, contributing to horizontal transmission among animals within and between flocks, there is still a distinct knowledge gap in effective management of this route compared to others, like direct contact, fomite, or litter-mediated. This literature review has shown that aerosol transmission is governed not only by the nature of the respiratory pathogens involved but also by broader biological processes – including particle behavior, environmental stressors, pathogen biology, and housing systems. In general, aerosol transmission is a major concern in high-stock-density poultry facilities, as these systems create more favorable conditions for the recirculation of bioaerosols generated by birds’ activities and housing management practices, such as feed handling, ventilation, litter disturbance, and manure accumulation (Cambra-López et al., 2010; Nguyen et al., 2022; Pereira et al., 2023; Górny et al., 2023). Major mitigation strategies include housing environment management, such as air filtration, humidity control, and efficient ventilation, to reduce aerosol exposure (Kovács et al., 2025; Van Seventer and Hochberg, 2016). Given these conditions, aerosol exposure in intensive poultry systems requires greater research attention and regular monitoring.

Current studies have several limitations regarding aerosol transmission that must be considered and addressed in future research. First, most molecular methods in related studies focus on the investigation of DNA or RNA, which does not always confirm the viability of microorganisms. At the same time, the methods for air sampling, particle sizing, and viability assessment results remain difficult to compare across studies and pathogens. In addition, while aerosol experiments and isolation studies are useful for elucidating general concepts, they unfortunately do not fully capture the complex interactions and environmental variability found in commercial houses. Information related to infectious dose, aerosol persistence under practical farm conditions, and long-term farm-scale validation is also still limited for many respiratory pathogens. In addition, low producer participation and compliance further hinder the implementation of monitoring and intervention efforts on commercial farms, limiting the availability of real-time, practical field data that can be effectively used for research purposes. These overall gaps emphasize the need for further research on methodological standardization in aerosol transmission research. Future studies should focus on implications for intensive commercial poultry production, with a priority on pathogen load, particle size distribution, and environmental variables such as temperature, humidity, and airflow (Yang et al., 2025; Wang et al., 2025; Xu et al., 2022). Advancements in aerosol monitoring, metagenomics, molecular diagnostics, and source-tracking tools for aerosols may further enhance understanding of aerosol transmission pathways (Kistanova et al., 2024; Schiappacasse et al., 2020). Finally, the aerosol route is also a concern for several zoonotic diseases, such as avian influenza and Newcastle disease, as poultry house workers are directly at risk. Avian influenza has greater zoonotic potential because its severity depends on the virus strain, which affects human health throughout the poultry production chain from farm to fork. Further studies and control measures related to zoonotic transmission via the aerosol route should also be considered for human health.

## 7 Conclusion

Aerosol transmission is a significant pathway in the spread of infectious diseases in poultry. Its importance lies not only in the movement of pathogens but also in its interactions with animals and the immediate housing environment – which, in itself, reflects management quality – that influence host exposure and disease susceptibility. Despite its multi-faceted nature, there still remains a challenging knowledge gap in current literature regarding discussions of the aerosol transmission route – namely a lack of a holistic approach to its study and management. Only by integrating these various factors governing both host and pathogen behaviors will researchers and producers be able to develop a comprehensive understanding of this transmission route and its associated nuances, on which effective management and preventive strategies can be developed. Ultimately, these efforts will contribute to improved animal health, welfare, and productivity, ensuring sustainability in the poultry industry and its goals of providing the ever-growing global population with a source of affordable, high-quality animal protein.
